# Influence of Renewable Nano-Al_2_O_3_ on Engine Characteristics and Health Impact under Variable Injection Timings and Excess Air Coefficients

**DOI:** 10.1021/acsomega.4c09313

**Published:** 2024-12-06

**Authors:** Zhefeng Guo, Yu-Lun Hsieh, Sheng-Lun Lin, Yen-Yi Lee, Timothy H. Lee

**Affiliations:** † College of Energy Engineering, Zhejiang University, Hangzhou 310027, PR China; ‡ ZJU-UIUC Institute, Zhejiang University, Haining 314400, PR China; § Department of Environmental Engineering, National Cheng Kung University, Tainan 70101, Taiwan; ∥ Institute of Environmental Toxin and Emerging Contaminant, Cheng Shiu University, Kaohsiung 83347, Taiwan; ⊥ Center for Environmental Toxin and Emerging-Contaminant Research, Cheng Shiu University, Kaohsiung 83347, Taiwan

## Abstract

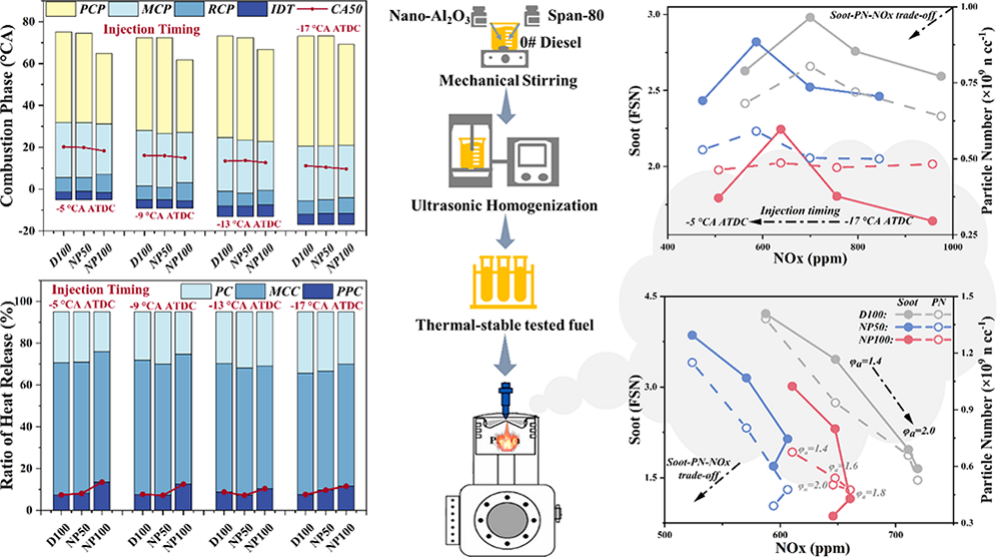

Nano-Al_2_O_3_ derived from recyclable sources emerges as a promising sustainable solution for enhancing diesel engine efficiency while mitigating emissions. However, a lack of an in-depth understanding of the health hazard aspect still challenges its commercial applications. To this end, nano-Al_2_O_3_/diesel (NAD) blends prepared via ultrasonic homogenization were experimentally and analytically investigated under various injection timings and excess air coefficients to explore the potential of nano-Al_2_O_3_ for balancing energy performance and emissions. Results revealed a synergistic effect between the NAD blends and optimized combustion control strategies. NAD blends presented enhanced heat release and pressure rise rates even under late injection or hypoxic conditions, indicating a faster and more complete combustion. Specifically, NAD blends promoted the partially premixed combustion phase and reduced postcombustion duration. While a slight increase in fuel consumption and a decrease in thermal efficiency were observed, potentially due to minor chamber compatibility issues, a significant improvement in emissions was identified. NAD blends effectively mitigated the well-known soot-particulate number-nitrogen oxide (NOx) trade-off inherent in diesel engines. NAD blends achieved lower NOx emissions through the even temperature distribution promoted by nano-Al_2_O_3_, minimizing the formation of NOx precursors. Simultaneously, NAD blends contributed to a reduction in soot emissions as well as an increment in nucleation mode particles, which are smaller and more harmful than conventional engine-out particulates. Notably, deposition modes highlighted that a higher nano-Al_2_O_3_ addition leads to an increase in nucleation mode particles, resulting in a higher alveolar deposition (*d*_p_ = 5–100 nm) and lower nasal deposition (*d*_p_ = 200–800 nm). These findings suggest that, by optimizing injection timing and excess air coefficients, NAD blends offer a promising approach to enhance combustion and achieve cleaner emissions simultaneously, making them a valuable contribution to the development of more sustainable diesel engine technologies.

## 1 Introduction

Diesel engines (DEs) are integral to transportation, power generation, and mining sectors due to their high thermal efficiency and cost-effectiveness. However, their continued viability hinges on addressing environmental concerns. Stringent regulations target nitrogen oxide (NOx) and particulate matter (PM) emissions from DEs and prompt extensive research efforts. These efforts encompass fuel modification^1^ and multifuel systems,^2^ combustion control strategies,^3^ combustion chamber modifications,^4^ and advanced after-treatment technologies.^5^ Among these approaches, fuel additives present a particularly attractive solution. They have the potential to simultaneously mitigate challenges posed by dwindling fossil fuel reserves, increasingly stringent emission regulations, and the demand for improved engine efficiency.^6^

DEs face the critical challenge of balancing efficiency with environmental sustainability. Multicomponent fuels have emerged as a promising solution, enhancing thermal efficiency and environmental compatibility by promoting improved in-cylinder combustion.^7−9^ The utilization of recirculated hydrous solvents and waste oil as DE fuel additives holds particular appeal.^10^ These alternatives not only contribute to improved performance but also potentially reduce net carbon emissions by diverting waste streams from landfills.^11^ Meanwhile, the advent of nanotechnology has shifted focus toward nanoparticles (NPs) as potential fuel additives, owing to their exceptional specific surface area and thermal conductivity, which are shown to enhance engine performance and curb toxic emissions.^12−15^ Furthermore, NPs are shown to significantly boost the thermodynamic performance of DEs.^16,17^ Kegl et al.^13^ highlight that among various NPs, nano-Al_2_O_3_ particularly stands out for its ability to refine DE performance through its high thermal conductivity, impacting both energy performance and emission reduction. Nano-Al_2_O_3_ also shows promise in improving cold start capabilities by enhancing diesel droplet ignition at low temperatures, addressing a critical limitation of conventional DEs.^18^

Al_2_O_3_ is one of the main recyclable components in many wastes. Its recycling pathways are diverse, offering both resource circularity and environmental benefits. Industrial waste slags, such as those from electric arc furnace steelmaking, contain significant amounts of Al_2_O_3_ and can be processed by using physical or chemical methods to recover the Al_2_O_3_ content. Techniques such as magnetic separation, flotation, and acid leaching are commonly used. Similarly, spent catalysts in the petrochemical industry can be regenerated through calcination, acid washing, or chemical processing to recover Al_2_O_3_ or restore catalytic activity. Nevertheless, Al_2_O_3_ is also present in inorganic sludges generated from water treatment and mineral processing, where it can be extracted through chemical precipitation and centrifugal separation. These processes highlight the potential for the production of nano-Al_2_O_3_ through recycling.

Extensive studies have explored the effects of nano-Al_2_O_3_ concentration and particle size on combustion, energy efficiency, and emissions under various engine operational parameters.^19−36^ These investigations confirm that nano-Al_2_O_3_ reduces emissions and promotes complete combustion by enhancing evaporation and heat transfer.^29−31^ Nonetheless, while there is evidence of nano-Al_2_O_3_ influencing in-cylinder air-fuel mixing,^28^ limited research exists on its specific impact on combustion phases and interactions with combustion control strategies like injection timing (IT) and excess air coefficient (φ_a_). Prior studies highlight a synergistic potential between NPs and such strategies, enhancing engine performance through precise IT adjustments.^37−40^ Low φ_a_ results in an overly rich mixture within the combustion chamber, precipitating incomplete combustion that adversely impacts PM emissions and the economic efficiency of DEs. Notably, nano-Al_2_O_3_ has demonstrated the ability to create more homogeneous air-fuel mixtures,^20,21^ making it imperative to investigate its role under conditions characterized by late injection or hypoxic conditions.

In addressing toxic emissions from DEs, the role of nano-Al_2_O_3_ additives emerges as critical. These additives are shown to significantly mitigate gaseous carbon emissions such as carbon monoxide and unburned hydrocarbons,^23−26^ resulting from incomplete combustion, aligning with the global push toward carbon neutrality. However, introducing nano-Al_2_O_3_ may inadvertently increase NOx emissions due to its capacity to reduce the ignition delay and accelerate combustion, leading to faster heat release and elevated pressure within the combustion chamber.^29^ Nonetheless, nano-Al_2_O_3_ also has the potential to counteract NOx formation by enhancing heat transfer and reducing localized overheating,^30^ indicating a nuanced interplay affecting NOx emission trends.

PM and particulate number (PN) emissions, significant concerns in DEs, primarily caused by uneven air-fuel mixing, are also influenced by nano-Al_2_O_3_ additives. Particle categorization by particle diameter (*d*_p_) includes nucleation mode (NM, *d*_p_ < 50 nm), accumulation mode (AM, 50 < *d*_p_1000 nm), and coarse mode,^41^ with DE emissions primarily comprising NM and AM particles. Studies, including those by Venu et al.,^19,20^ Fayad et al.,^21^ and Dhahad et al.,^24^ have demonstrated the capability of nano-Al_2_O_3_ to reduce PM emissions by inhibiting the formation of PM precursors. Concurrently, a clear decreasing trend in *d*_p_ is observed with the introduction of nano-Al_2_O_3_.^19^ Furthermore, an increase in PN emissions has been reported, attributed to a rise in the concentration of NM particles.^42,43^ This rise is problematic, particularly given the decreased efficiency of diesel particle filters (DPF) in capturing particles smaller than 50 nm, allowing a significant number of NM particles to bypass the DPF system.^44^ Unfortunately, these escaping inhalable particles (aerodynamic diameter ≤10 μm) can deposit in the human respiratory system via various fluid dynamics mechanisms, especially those NM particles with a deposition rate >50%.^45^ Furthermore, Heyder et al. measured the deposition rates of particles with a range of 0.005–15 μm throughout the human respiratory system under oral and nasal breathing, as well as the deposition rates of nasal, laryngeal, bronchial, and alveolar regions.^46^ Results manifested that in the range of DE emissions, a finer *d*_p_ features a higher deposition rate. Therefore, a detailed examination of NM and AM particle distributions and their deposition is crucial, extending beyond the total PM and PN concentrations.

This backdrop highlights nano-Al_2_O_3_ as a potential and effective fuel additive for DEs, offering the dual benefits of improved fuel efficiency and reduced emissions. However, the detailed effects of nano-Al_2_O_3_ on in-cylinder combustion dynamics and environmental and health hazards under challenging air-fuel mixing scenarios require further exploration. This study aims to comprehensively investigate nano-Al_2_O_3_’s viability as a fuel additive for DEs, focusing on in-cylinder combustion dynamics, energy efficiency, emission profiles, and the PN deposition of various nano-Al_2_O_3_/diesel blends. By conducting an in-depth analysis, this study seeks to elucidate the complex balance between enhancing engine performance and minimizing environmental and health impacts, thereby contributing to the sustainable development of diesel engine technology.

## 2 Experimental Method

### 2.1 Fuel Preparation

The base fuel utilized in this study was conventional 0# diesel. Nano-Al_2_O_3_, specifically γ-Al_2_O_3_ with an approximate particle diameter of 30 nm, was selected for its favorable properties. It is important to note that diesel and nano-Al_2_O_3_ are inherently immiscible owing to their differing physical phases. This immiscibility can lead to the agglomeration of nano-Al_2_O_3_ particles when dispersed in diesel, driven by van der Waals forces. Such agglomeration diminishes the specific surface area of nano-Al_2_O_3_, negatively impacting the thermal transfer characteristics of the nano-Al_2_O_3_/diesel (NAD) blend.

Addressing this challenge, prior research has highlighted that the application of surfactants and ultrasonic homogenization techniques can significantly enhance the stability of nanoparticle dispersion in diesel, extending the duration of stable dispersion.^47,48^ In this context, NAD blends with concentrations of 50 and 100 mg/L (denoted as NP50 and NP100, respectively) were prepared, each incorporating 0.5 and 1 vol % of the surfactant Span-80. Further, the NAD blends underwent a 20 min treatment using an ultrasonic homogenizer equipped with a 20 mm amplitude transformer (maximum power 1200 W, frequency 19.5–20.5 kHz), set to a power of 1080 W. This device was programmed to pause for two s after every two seconds of operation, ensuring the diesel temperature remained below 45 ± 2 °C throughout the process. The NAD blends demonstrated thermal stability exceeding 72 h without evidence of stratification, indicating successful dispersion. To mitigate potential effects from nano-Al_2_O_3_ agglomeration, experiments were conducted promptly after the blending process.

### 2.2 Diesel Engine Experimental Setup

**Figure 1:**
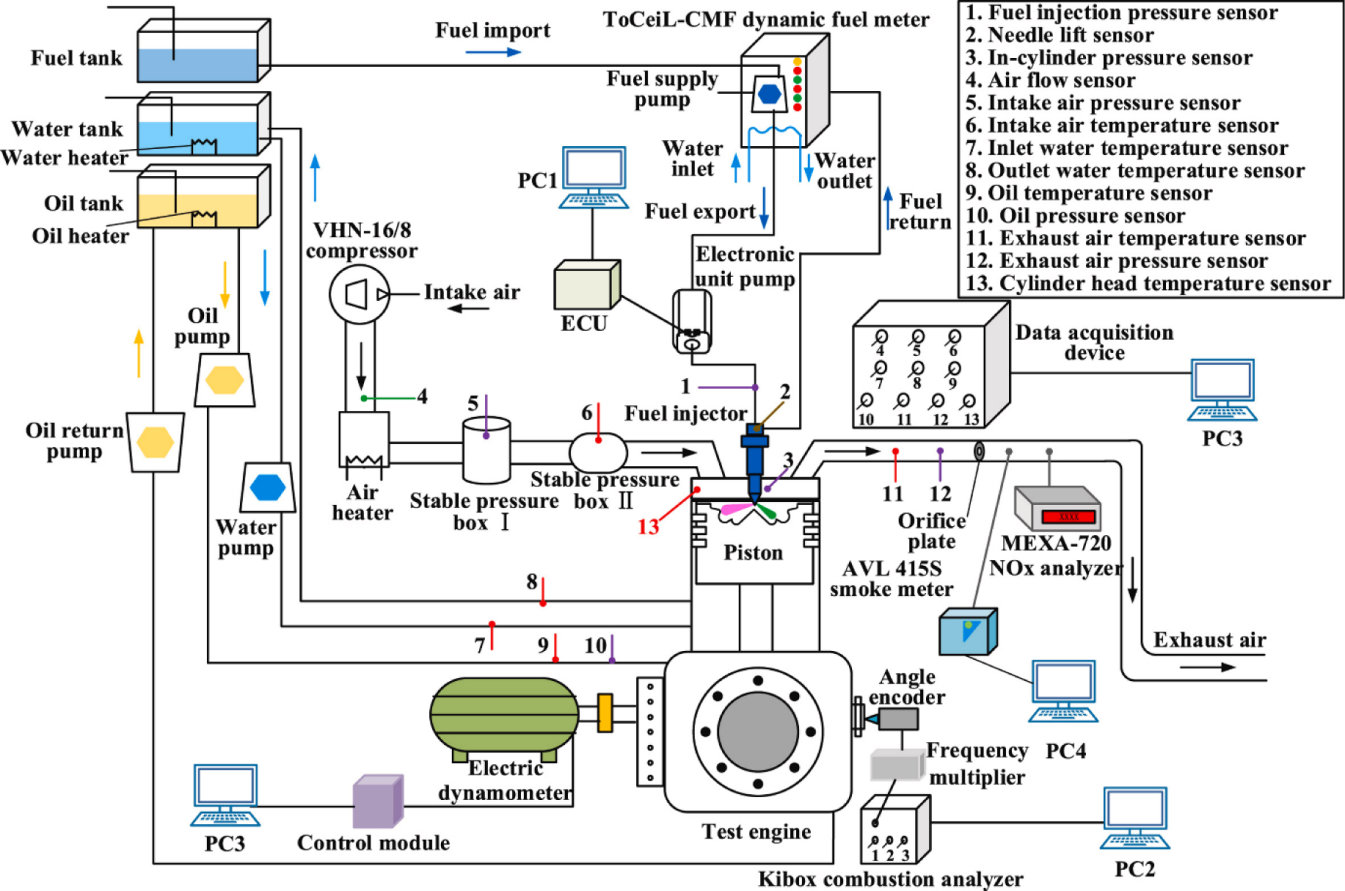
Schematics of testing the single-cylinder DE and sampling/monitoring system.

In this investigation, a turbocharged single-cylinder diesel engine (DE) setup was utilized for conducting a series of tests to evaluate the in-cylinder combustion dynamics, energy efficiency, and emission profiles when operating on pure diesel and NAD blends. Detailed specifications of the DE are enumerated in Table 1, and a comprehensive schematic representation of the DE setup, inclusive of the sampling system, is depicted in Figure 1.^4^

**Table 1:** Specifications of the Employed Single-Cylinder DE

Items	Specifications
Bore × stroke (mm)	132 × 145
Compression ratio (−)	13.5
Rated speed and power (r/min and kW)	2500, 92
Nozzle number × diameter (mm)	8 × 0.27
Intake valve opening and closure (°CA)	347 and –123
Exhaust valve opening and closure (°CA)	118 and –356

The engine setup featured an electric dynamometer with a maximum capacity of 160 kW and an operational speed of 4500 rpm. For fuel delivery, a high-precision Bosch electronic unit pump was employed, characterized by a plunger diameter of 12 mm, a prestroke measurement of 9 mm, and a peak injection pressure reaching 160 MPa. To accurately replicate the conditions of turbocharging and intercooling, the system incorporates an electronic compressor and an intake heater. Additionally, to mimic the exhaust back pressure typical of multicylinder engines, a throttle orifice has been integrated into the exhaust conduit.

Operational metrics, such as the fuel consumption rate, were meticulously monitored using a transient fuel consumption meter, boasting a response latency of less than 0.1 s. The transient combustion analyzer (Kibox) recorded the thermodynamic and injection parameters within the cylinder. Emissions of NO_x_ were quantified by employing a fast NO_x_ analyzer (Cambustion CLD500), which ensures minimal zero-point drift (under 5 ppm per hour) and a swift T10–90% response time of merely 2 ms. Particulate matter in the form of soot was measured using an AVL 415S smoke meter, offering an accuracy within ±0.2 filter smoke number (FSN). For PN emissions, a fast particle analyzer (Cambustion DMS500) was utilized, capable of a data sampling rate of 10 Hz and a T10–90% response time of 200 ms, enabling the detailed analysis of particulate emissions

The pressure and temperature of intake air, exhaust gas, and engine oil and the temperature and flow rate of the coolant were analyzed by a steady-state data collector. The accuracy of the measuring instruments is given in Table 2. Additionally, during the experiment, the intake air temperature, diesel temperature, engine oil temperature, and coolant temperature were controlled at 60 ± 2 °C, 40 ± 2 °C, 70 ± 5 °C, and 80 ± 5 °C, respectively.

**Table 2:** Accuracy of Measuring Instruments

Measuring Instruments	Accuracy	Notes
Electric dynamometer	Torque: ±0.3%FS	
	Speed: ±2r/min	
YTZ-150 pressure sensor	±1.6% FS	Intake/Exhaust air pressure
Kistler type 6052C pressure sensor	±0.5% FSO	In-cylinder pressure
K-Type thermocouple	±10°C	Exhaust air temperature
Pt100 thermal resistor	±0.3 °C	Intake air temperature
PT100 thermal resistor	±0.5% FSO	Oil temperature
Air flow sensor	±1% FSO	
Transient fuel consumption meter	0.12% FS	

### 2.3 Operational Conditions and Parameter Definitions

This study explored the impact of nano-Al_2_O_3_ particles on the in-cylinder combustion processes, energy efficiency, and emission outputs of diesel engines across varying injection timings (IT) of −5, −9, −13, and −17 crank angles after top dead center (°CA ATDC) and excess air coefficients (φ_a_) set at 1.4, 1.6, 1.8, and 2.0. φ_a_ was regulated via the aforementioned transient fuel consumption meter, while the IT adjustments were made through the engine’s electronic control unit. The quantity of fuel injected was meticulously controlled according to the engine’s load requirements, dictated by the injection timing and duration parameters. It is crucial to acknowledge that the oil tank was scrubbed and the engine was operated for 30 min with the newly introduced test fuel to eliminate any potential contamination from previously used fuels. To ensure the reliability of the experimental data, each test condition was replicated three times.

The evaluation of in-cylinder thermodynamic parameters, recorded by the transient combustion analyzer, enabled segmentation of the combustion process into distinct phases: ignition delay time (IDT), rapid combustion phase (RCP), main combustion phase (MCP), and postcombustion phase (PCP). These phases are delineated as follows: IDT spans from the onset of injection timing to the completion of initial ignition; RCP extends from the start of initial ignition to the conclusion of the first significant decrease in the heat release rate (HRR); MCP progresses from the end of this decrease in HRR to the peak of the in-cylinder temperature; finally, PCP encompasses the period from this peak temperature to the point where 95% of the cumulative HRR (CA95) has been achieved. Moreover, to dissect the heat release rate (HRR) pertinent to each combustion phase, the total heat contribution is divided into partially premixed combustion (PPC), mixing-controlled combustion (MCC), and postcombustion (PC) segments. Here, PPC accounts for the HRR during the RCP, MCC covers the HRR during the MCP, and PC pertains to the HRR during the PCP.

Considering the range of *d*_p_ emitted from diesel engines and the correlation between deposition rates in the human respiratory system and *d*_p_, this study analyzed total, nasal, and alveolar particle deposition. Given that nasal breathing is more common, a nasal breathing deposition rate was employed with an average flow rate of 750 cm^3^/s, a respiratory cycle of 4 s, and a tidal volume of 1500 cm^3^.^46^ Additionally, cubic spline interpolation performed using MATLAB was used to obtain the total, nasal, and alveolar deposition rates and PN in the range of 5–1000 nm. In this context, the deposition PN in the human respiratory system was calculated using the following equation:
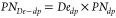
1where *De*_dp_ represents the total, nasal, and alveolar deposition rates under specific *d*_p_ and where PN_*dp*_ represents the PN under specific d_p_.

## 3 Results and Discussion

### 3.1 Impact of Nano-Al_2_O_3_ on Diesel Engine In-Cylinder Combustion Characteristics

#### 3.1.1 Heat Release Rate and Pressure Rise Rate

The analysis of the heat release rate (HRR) provides insights into the in-cylinder combustion dynamics and thermal energy released per unit of the crank angle (°CA), serving as a pivotal indicator of the combustion efficiency within the diesel engine (DE). On the other hand, the pressure release rate (PRR) is indicative of combustion intensity and the dynamic pressure conditions within the cylinder. A heightened PRR peak signifies a more rapid and intense combustion process.

**Figure 2:**
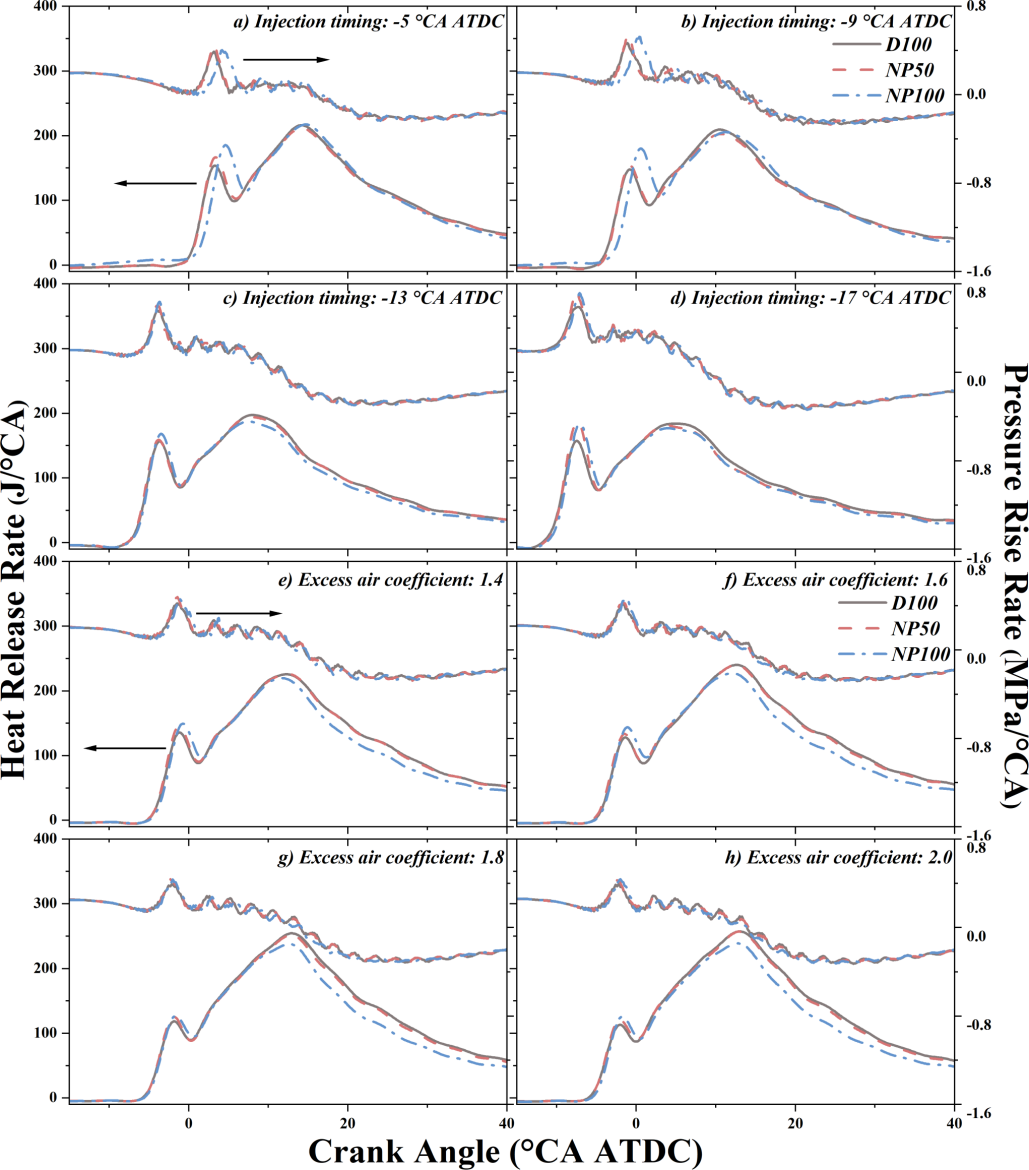
Effects of nano-Al_2_O_3_ on heat release and pressure rise rates at various (a–d) injection timings and (e–h) excess air coefficients.

As depicted in Figures 2a–d, with the advancement of injection timing (IT), peaks associated with partially premixed combustion (PPC) and mixing-controlled combustion (MCC) occur earlier in the HRR curve. This advancement is attributed to a reduction in ignition delay time (IDT), enhancing the combustion rate. The incorporation of nano-Al_2_O_3_ notably heightens the peak of PPC, a trend that intensifies with increasing concentrations of nano-Al_2_O_3_, corroborating existing studies on its benefits in air-fuel mixing due to its superior thermal conductivity and specific surface area.^34^ The effect is particularly pronounced for NP100, which, at −17 °CA ATDC, exhibits a shorter IDT and achieves a more homogeneous air-fuel mixture, culminating in a significant rise in the PPC peak.

Exploring the effects of nano-Al_2_O_3_ on HRR at various excess air coefficients (φ_a_) indicates that for both pure diesel and NAD blends, the MCC peak escalates with higher φ_a_ (see Figures 2e–h). This elevation is linked to the increased oxygen content and the consequent acceleration of the combustion rate. Furthermore, an increase in φ_a_ not only precipitates the MCC peak sooner but also enhances its magnitude. The PPC peak similarly rises with higher concentrations of nano-Al_2_O_3_, echoing findings from previous studies.^19^ However, this increase in the intensity of the PPC peak is accompanied by a reduction in the intensity of the MCC peak, potentially detracting from the overall heat release. The specific reasons for the decline in MCC will be discussed in Section 3.2.

Across experimental conditions, the changing trend of PRR is highly correlated with the trend of HRR. The addition of nano-Al_2_O_3_ further amplifies PRR, due to improved heat transfer, which facilitates quicker heat release. A noteworthy observation is the unique behavior of NP100 at −5 °CA ATDC, where it registers a higher HRR but a lower PRR compared to NP50. This is due to the delayed peak HRR, which postpones the surge in the in-cylinder pressure, thereby moderating the PRR. Investigations into PRR dynamics reveal an initial decrease followed by an increase as φ_a_ rises, with excess air contributing to a more uniform mixture and a faster combustion rate. Moreover, the shortened IDT associated with excess air leads to an enhanced MCC, thereby modifying the combustion rate. The introduction of nano-Al_2_O_3_ significantly enhances heat transfer during combustion, promoting faster combustion rates, which in turn results in a more concentrated heat release and elevated PRR. Consequently, the addition of nano-Al_2_O_3_ consistently leads to an increase in PRR.

#### 3.1.2 Combustion Phase, CA50, and Heat Release Ratio

**Figure 3:**
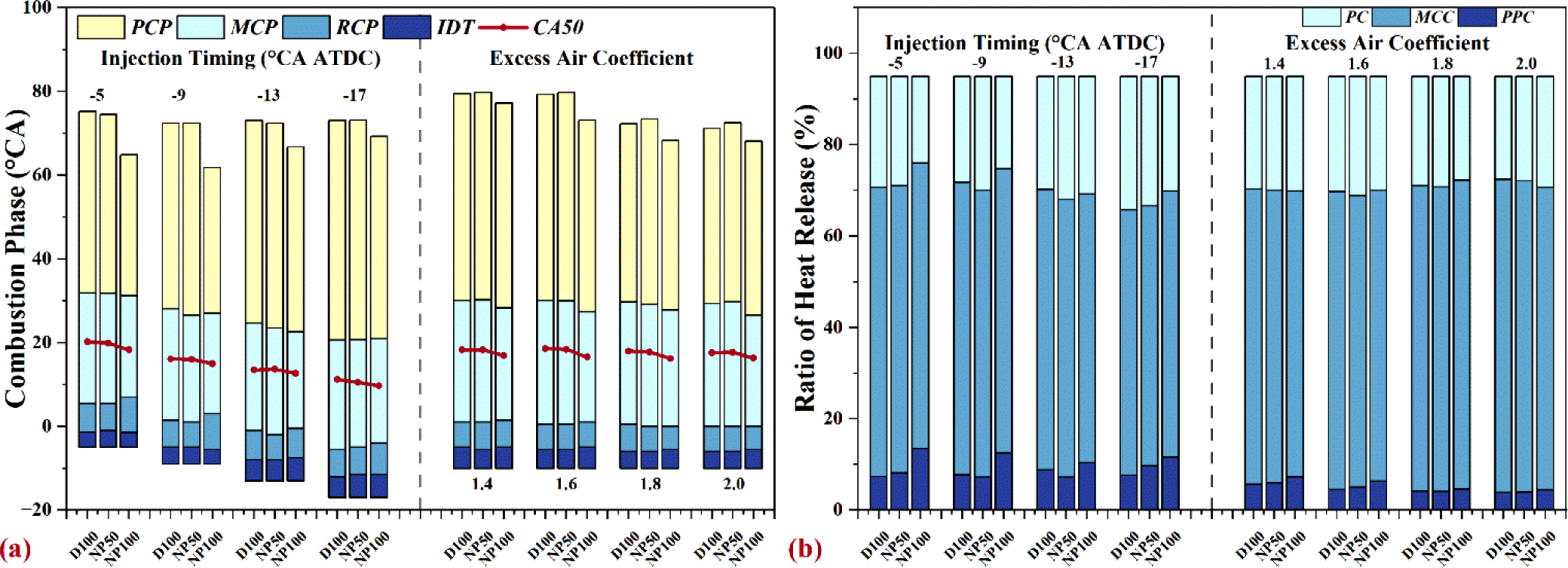
Effects of nano-Al_2_O_3_ on (a) the combustion phase and CA50 and (b) the ratio of heat release at various injection timings and excess air coefficients.

Figure 3a elucidates the influence of nano-Al_2_O_3_ on the combustion phase and the crank angle at 50% heat release (CA50) under various IT and φ_a_ conditions. A notable observation is the reduction in IDT as the IT is delayed. This alteration significantly shortens the combustion phase and advances the CA50, a phenomenon attributable to the accelerated combustion pace brought about by the shortened IDT.^49^ Additionally, the integration of nano-Al_2_O_3_ further reduces the IDT and combustion duration time (CDT), advancing CA50, particularly with NP100. This outcome is largely due to nano-Al_2_O_3_’s enhancement of heat transfer, expediting combustion processes.^19,23,34^ The observed reduction in CDT is predominantly a result of the abbreviated postcombustion phase (PCP). Specifically, at an IT of −5 °CA ATDC, NP100 exhibits a CDT of 69.9 °CA, which is 10.3 °CA shorter than that of D100, with its PCP being 33.7 °CA, 9.6 °CA shorter than that of D100. In addition, in the context of pure diesel and NAD blends, an increment in φ_a_ leads to a marginal advancement in the CA50 and a reduction in the CDT, corroborating the research findings by Li et al.^4,50^ The rationale behind this trend is the higher oxygen concentration within the combustion chamber, stemming from the elevated φ_a_, which, in turn, shortens the IDT and accelerates the combustion process. Interestingly, although NAD blends do advance CA50, the impact on CDT varies with the concentration of nano-Al_2_O_3_. NP50 shows a slight increase in CDT, whereas NP100, particularly at lower φ_a_ levels, significantly accelerates combustion, resulting in a markedly reduced CDT.

Figure 3b details the impact of nano-Al_2_O_3_ on the ratio of heat release (RHR) under varying IT and φ_a_ conditions. Delaying the injection time amplifies the heat release contributions from both PPC and MCC. The addition of nano-Al_2_O_3_ further elevates the heat release contributions from PPC and MCC, with the extent of this enhancement growing with the concentration of nano-Al_2_O_3_. For instance, at an injection timing of −5 °CA ATDC, the contribution of postcombustion (PC) to the overall heat release amounts to 24.3% for D100, 24.0% for NP50, and 19.0% for NP100. Generally, a diminished contribution from PC to the total heat release signifies a more complete and rapid combustion process, beneficial for the engine’s efficiency.^34^ Increasing φ_a_ also tends to decrease the contribution of PC to the total heat release by diminishing the PPC contribution while increasing that of MCC, as illustrated in Figure 3b. Across various φ_a_, the introduction of nano-Al_2_O_3_ enhances the PPC contribution but reduces that of MCC, indicating a shift in the combustion dynamics of the NAD blends.

These phenomena manifest that the presence of nano-Al_2_O_3_ aids in minimizing the heat release contribution from PC, particularly under low φ_a_ or late injection scenarios, where richer air-fuel mixtures are prevalent. This beneficial outcome can improve the energy conversion efficiency under rich mixture conditions, attributed to nano-Al_2_O_3_’s promotion of faster and more intense combustion.

### 3.2 Impact of Nano-Al_2_O_3_ on Diesel Engine Energy Performance

**Figure 4:**
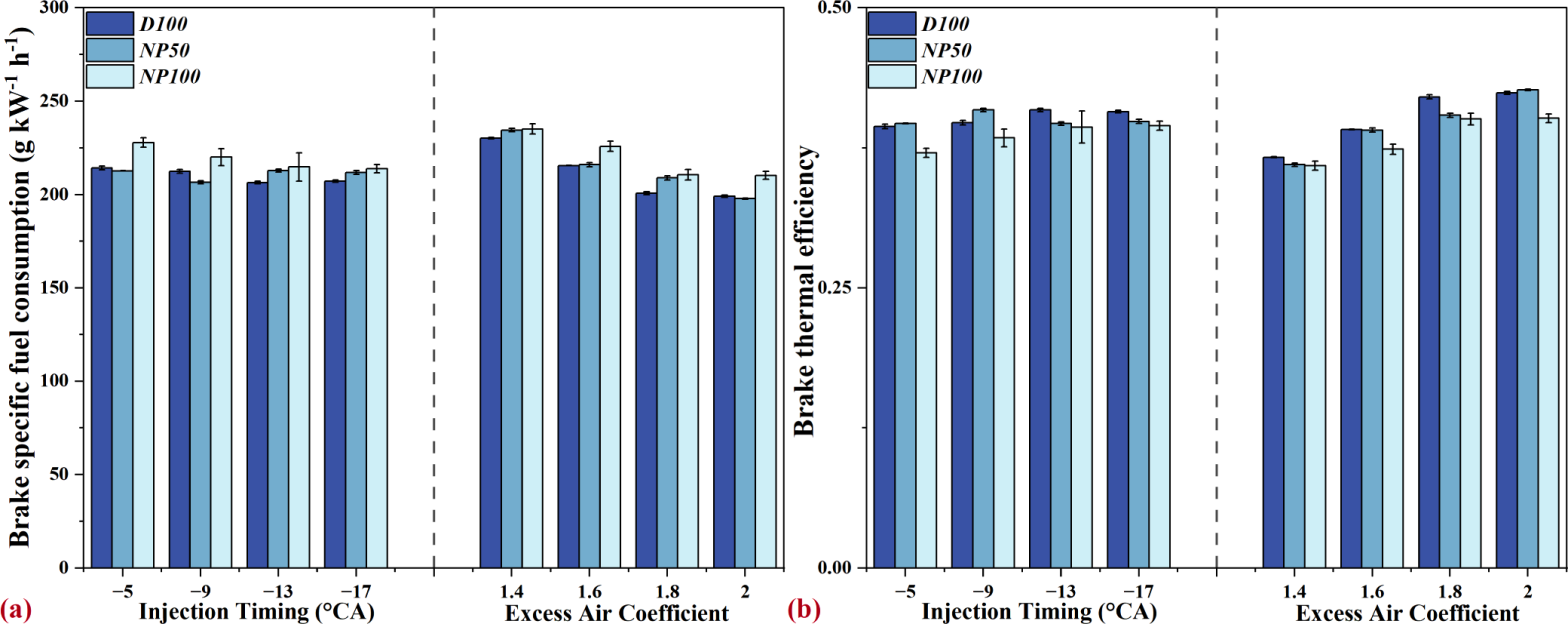
Effects of nano-Al_2_O_3_ on (a) brake specific fuel consumption and (b) brake thermal efficiency at various injection timings and excess air coefficients.

Energy performance of DEs is quantified through indicators such as brake-specific fuel consumption (BSFC) and brake thermal efficiency (BTE), as shown in Figure 4. The impact of varying IT and φ_a_ on the BSFC and BTE is complex. Delayed injection timing tends to diminish BTE and elevate BSFC due to poorer air-fuel mixing, resulting in incomplete combustion. Conversely, increasing φ_a_ typically enhances combustion completeness and efficiency, leading to an improvement in BTE and a reduction in BSFC. These dynamics, however, diverge from findings in earlier research,^27,29,32,33^ where the introduction of nano-Al_2_O_3_, under most conditions, slightly worsened BSFC and reduced BTE. This deviation is attributed to nano-Al_2_O_3_’s influence on the heat release process; while it increases the contribution from PPC, it concurrently reduces the contribution from MCC. Such alterations in the combustion dynamics lead to a lower overall heat release from NAD blends, adversely affecting BTE and increasing BSFC.

Further insights from our preceding investigation into the combustion chamber design (DSC98) of the engine used here shed light on the rationale behind this abnormal phenomenon,^51^ which uncovered a pronounced tendency for fuel to accumulate near the walls of the combustion chamber in DSC98 configurations. The presence of nano-Al_2_O_3_ exacerbates this issue by enhancing the kinetic energy of breakup droplets, owing to their high density, which in turn extends liquid spray penetration^52^ and worsens fuel accumulation. This increased fuel accumulation heightens the likelihood of incomplete combustion, further reducing the contribution of MCC, and ultimately worsening the BTE.

Moreover, the fuel liquid length escalates with higher concentrations of nano-Al_2_O_3_,^52^ intensifying the challenges associated with fuel accumulation. Consequently, the combustion chamber compatibility challenges need to be considered; otherwise, engines operating on NAD blends require a higher fuel input to achieve the same power output as those running on conventional diesel. This nuanced understanding emphasizes the intricate interaction among nanoparticle additives, combustion processes, and engine design factors in shaping the overall energy efficiency of diesel engines.

### 3.3 Impact of Nano-Al_2_O_3_ on Diesel Engine Pollutant Emissions

#### 3.3.1 Soot, NOx, and In-Cylinder Peak Temperature

**Figure 5:**
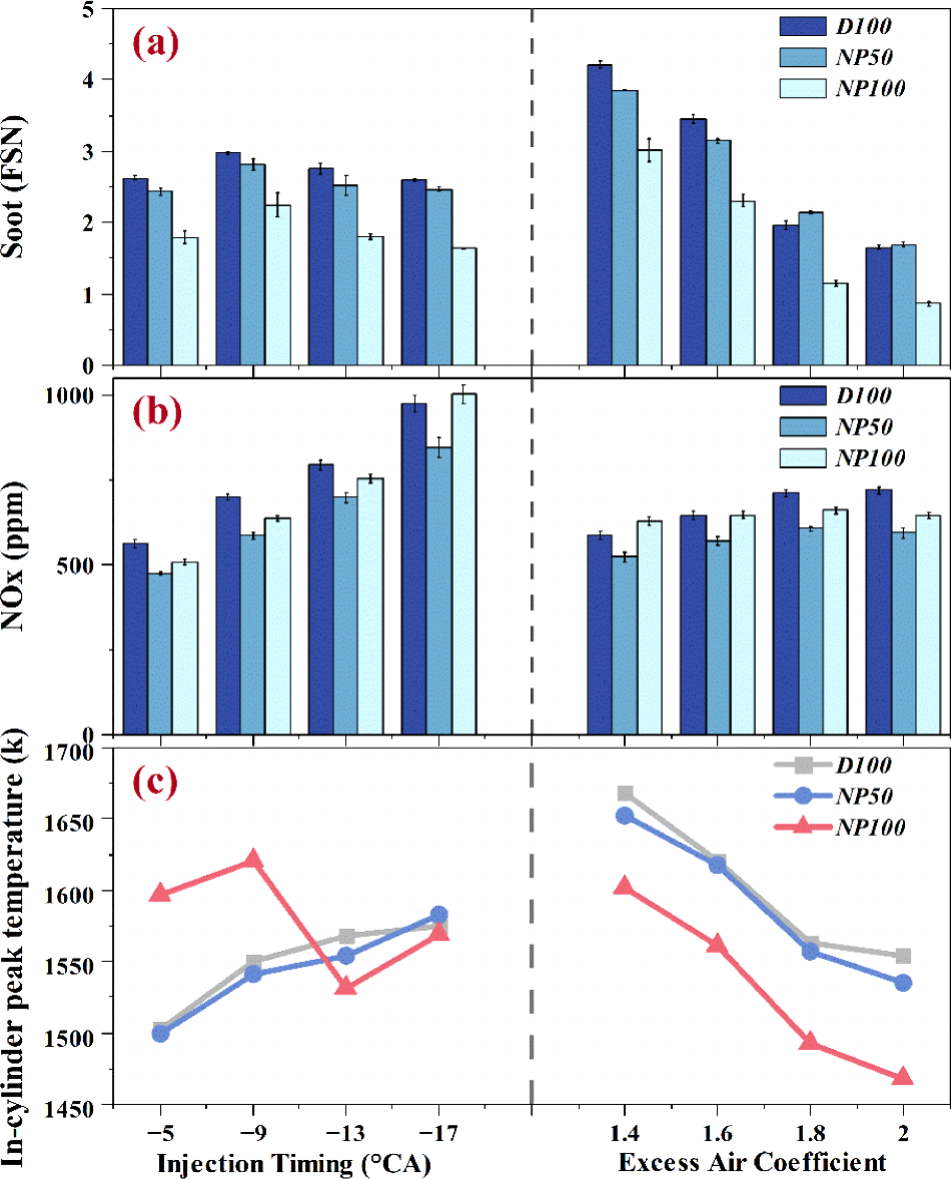
Effects of nano-Al_2_O_3_ on (a) soot emissions, (b) NOx emissions, and (c) the in-cylinder peak temperature at various injection timings and excess air coefficients.

The impact of IT and φ_a_ on soot emissions is depicted in Figure 5a. Late injection, occurring near the top dead center, typically hampers adequate mixing of air and fuel, thus elevating soot emissions.^49^ Contrary to expectations, this study found that soot emissions did not consistently increase with delayed IT. The highest soot emissions were recorded at −9 °CA ATDC, with increases for D100, NP50, and NP100 of 11.8%, 13.7%, and 20.1%, respectively, compared to levels at −5 °CA ATDC. Notably, increasing φ_a_ significantly reduces soot emissions; a φ_a_ increase from 1.4 to 2.0 resulted in a decrease in emissions from D100, NP50, and NP100 by 60.8%, 56.2%, and 71.3%, respectively. This reduction is attributed to excess air reducing in-cylinder temperatures and elevating oxygen concentration, which promotes soot oxidation.^20,33,53^ The introduction of nano-Al_2_O_3_ further diminishes soot emissions, with greater reductions observed at higher nano-Al_2_O_3_ concentrations. This is due to nano-Al_2_O_3_ providing additional reaction sites and enhancing the combustion of oxygen and fuel. Furthermore, nano-Al_2_O_3_ is also theorized to promote heat transfer, creating a more uniform combustion region and thereby inhibiting soot formation.^23^

The influence of nano-Al_2_O_3_ on NOx emissions and in-cylinder peak temperatures across different ITs and φ_a_ conditions is shown in Figure 5b,c. NOx formation in the chamber primarily follows the Zel’dovich mechanism, which is related to the in-cylinder temperature, represented by peak temperature in this study. This peak temperature refers to the maximum average temperature within the entire combustion chamber, typically occurring at the end of the diffusion combustion stage. Advancing IT and increasing φ_a_, which enhance combustion, results in higher in-cylinder temperatures and consequently increased NOx emissions. The effect of changing IT is more significant than that of φ_a_. Nano-Al_2_O_3_ helps reduce NOx emissions by enhancing heat transfer and mitigating local overheating within the combustion chamber.^34^ However, NP100 exhibits higher NOx emissions across a few experimental conditions, which are discussed in the following paragraph.

Notably, Figure 5c reveals that NP100 has a significantly higher peak temperature than D100 and NP50 at both −5 and −9 °CA ATDC, attributed to the enhanced heat release from premixed charge compression ignition (PCCI). Despite this, NP100 exhibits NOx emissions that are lower than those of D100. For instance, at −5 °CA ATDC, NOx emissions from D100 are 563 ppm, compared to 507 ppm from NP100, marking a 9.9% reduction. This suggests that nano-Al_2_O_3_ minimizes local overheating, allowing NP100 to maintain a higher in-cylinder temperature without the local temperature peaks associated with D100. Furthermore, despite nano-Al_2_O_3_ increasing both the PPC peak and PRR, NP50 marginally reduces the in-cylinder temperature, whereas NP100 significantly lowers it across multiple φ_a_. This phenomenon is mainly attributed to the lower cumulative HRR caused by fuel accumulation. Interestingly, NP100 exhibits higher NOx emissions than NP50, despite having a lower peak temperature. This observation, also evident across different IT settings, implies that the elevated PPC peak and PRR associated with NP100 lead to a more intense combustion process, thereby resulting in increased NOx emissions. Moreover, the advancement of the CA50 for NP100 extends the duration of interaction between oxygen and nitrogen under high-temperature surroundings, contributing to the increase in NOx emissions.

#### 3.3.2 PN Emissions and Deposition in the Respiratory System

**Figure 6:**
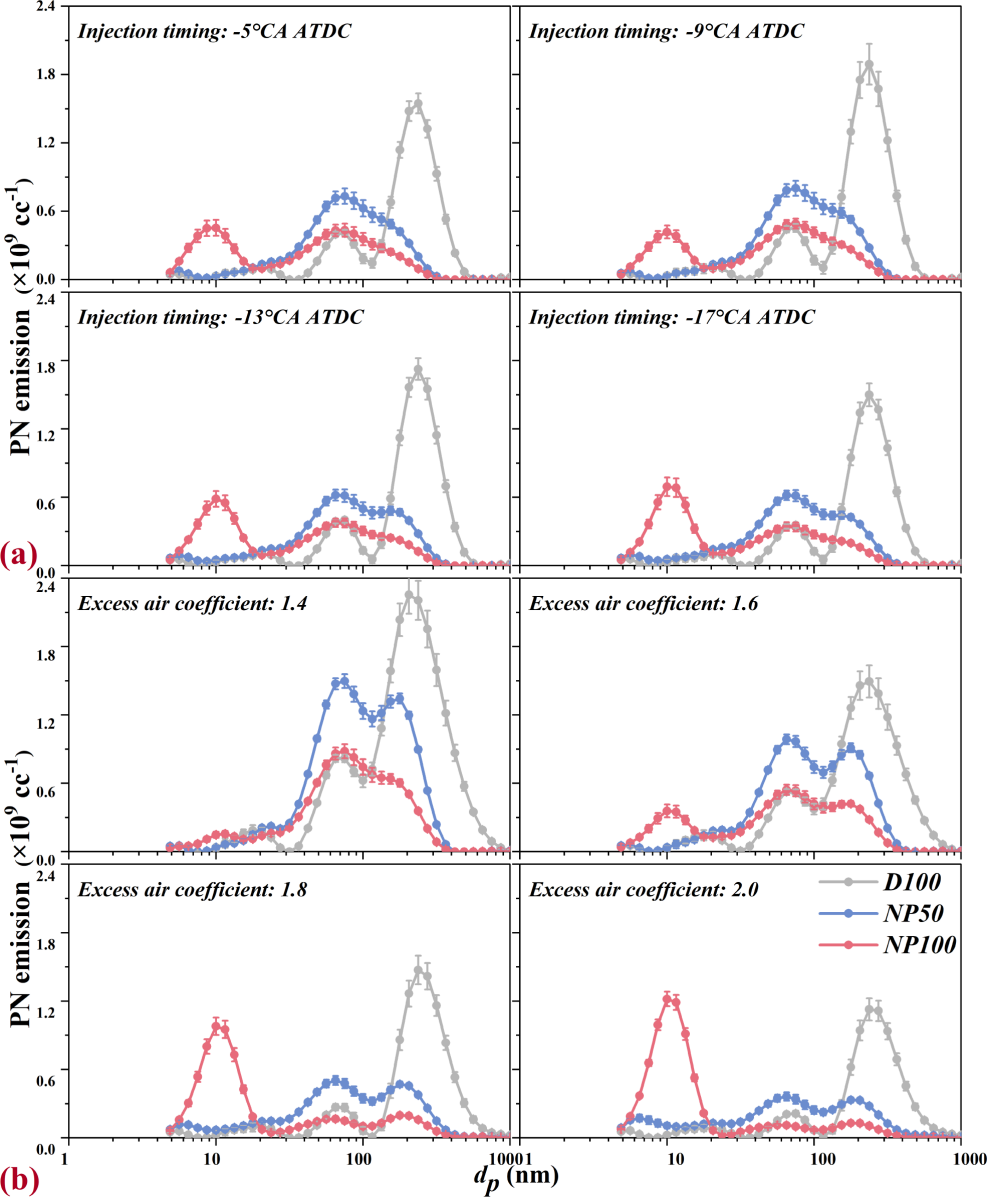
Effects of nano-Al_2_O_3_ on PN emissions at various (a) injection timings and (b) excess air coefficients.

The particle number-size distribution (PNSD) for D100 and NAD blends across various IT and φ_a_ conditions is detailed in Figure 6. Variations in IT slightly impact the PNSD characteristics for both D100 and NAD blends. D100 displays a consistent three-peak PNSD at different ITs, with peaks at approximately 10, 75, and 237 nm. In contrast, NAD blends exhibit altered distribution characteristics, notably lacking the large particle peak. Specifically, NP50 shows a near two-peak distribution, with peaks around 75 and 177 nm, while NP100s peak shifts toward finer particles, with a notable high concentration peak at about 10 nm. Alternatively, changes in φ_a_ distinctly affect PNSD characteristics. Elevated φ_a_ significantly reduces D100 and NP50s distribution peaks due to excess air mitigating local high temperatures and low oxygen areas, thus inhibiting particulate formation. For NP100, higher φ_a_ not only markedly decreases accumulation mode (AM) particles but also significantly boosts nucleation mode (NM) particles, with NP100s NM peak nearing D100s AM peak at φ_a_ of 2.0. NP50 also shows a tendency to produce more NM particles with increasing φ_a_, albeit to a lesser extent. This observation aligns with the findings by Lin et al., who noted an increase in KL factors, indicative of NM particles, appearing during combustion when nano-Al_2_O_3_ was introduced to diesel.^52^ Furthermore, near-wall combustion, resulting from fuel accumulation, is also an important cause of NM particles due to locally rich mixtures.^54^

**Figure 7:**
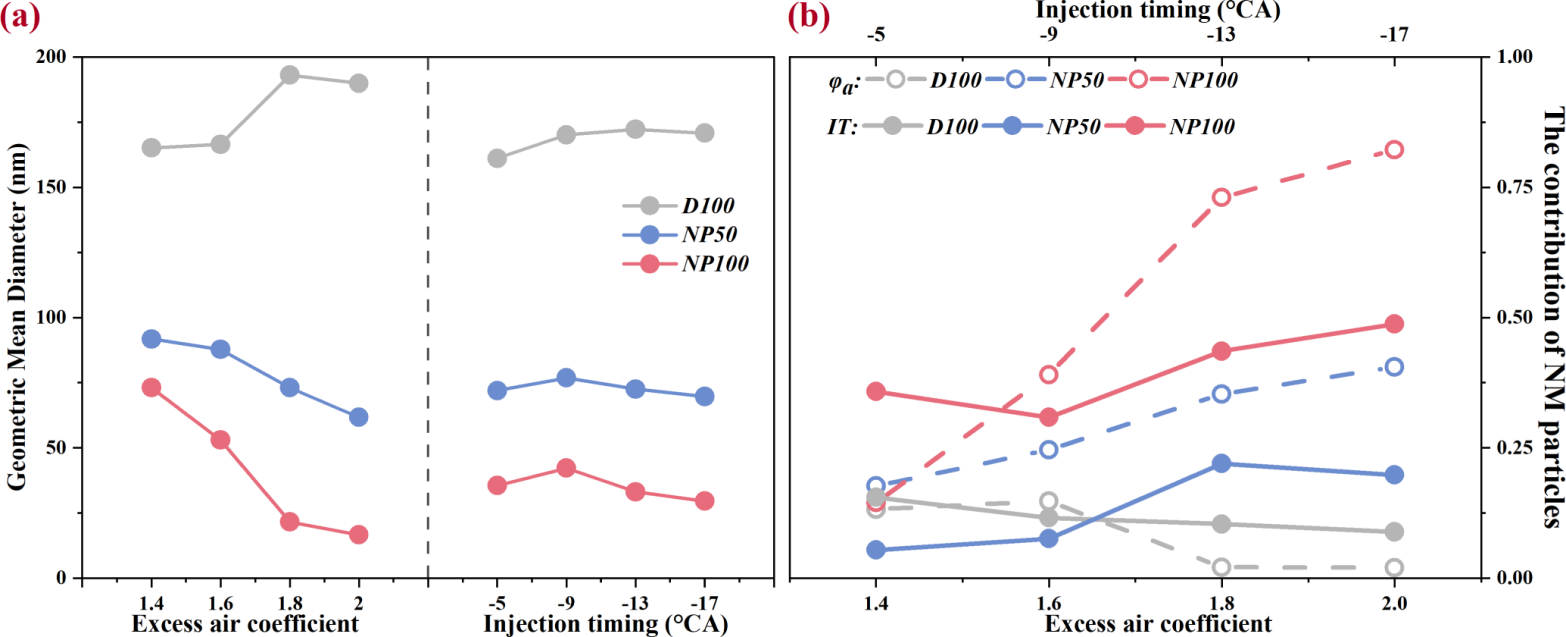
Effects of nano-Al_2_O_3_ on (a) GMD and (b) the contribution of NM particles under various conditions.

The statistical data of particle number (PN), including the geometric mean diameter (GMD) and NM particle concentration at various ITs and φ_a_ conditions, are further quantified in Figure 7. The NAD blends showed a decreasing GMD at high φ_a_ scenarios, whereas D100 exhibited an upward trend. This trend may be attributed to the role of excess air in lowering combustion temperatures, thereby increasing the likelihood of soot particle collision and agglomeration,^55^ which in turn elevates the GMD. Nonetheless, this effect is mitigated by the introduction of nano-Al_2_O_3_, which promotes the oxidation of larger particles into finer ones. Consequently, the most significant discrepancy in GMD between NAD blends and pure diesel is observed at a φ_a_ of 2.0, with the GMD of D100 reaching approximately 190 nm, in contrast to the substantially lower GMDs of NP50 and NP100, which stand at about 61.7 and 16.6 nm, respectively, marking reductions of 67.5% and 91.3%. At a φ_a_ of 2.0, the AM particle concentration was approximately 2% for D100, but it surged to 40.5% for NP50 and 82.3% for NP100. Aligning with PNSD, the GMD showcases a slight change with the advancement of IT. However, nano-Al_2_O_3_ introduction still significantly reduces the GMD, accompanied by a marked increase in NM particle concentration. For example, at −17 °CA ATDC, the GMD of NP50 and NP100 decreased by 56.8% and 79.3%, respectively, compared to D100. Meanwhile, the NM particle concentration of NP50 and NP100 increased to 19.8% and 48.7%, respectively. Notably, late injection or lower φ_a_, signifying rich mixture conditions, mitigates this adverse effect, aligning with the intended use of nano-Al_2_O_3_.

Moreover, previous studies have spotlighted the nano-Al_2_O_3_’s role in the PM formation process, which is diverged between D100 and NAD blends. According to the report, nano-Al_2_O_3_ provides additional reaction sites for adsorbing organic components and sufficient heat for their oxidation.^20^ The metal components in nanoparticles could agglomerate with soot precursors,^56^ inhibiting NM particle surface growth and condensation, leading to higher NM particle peaks in NAD blends. Additionally, previous studies have highlighted the nanoparticles’ role in rendering soot a sparse agglomerate structure with increased fringe separation distance, enhancing soot reactivity.^57^ Nanoparticles also lower the activation energy and oxidation temperature needed for soot particle oxidation.^56^ These findings suggest the nano-Al_2_O_3_’s potential in promoting soot oxidation. As φ_a_ increases, reducing unburned carbon and organic component concentrations, nano-Al_2_O_3_’s impact becomes pronounced. For NP100, this means inhibited soot surface growth and condensation alongside enhanced soot oxidation, making NM particles dominate NP100s PNSD. However, for NP50, due to its lower concentration of nano-Al_2_O_3_, the increase in NM particles is not as significant. It is crucial to explore different nano-Al_2_O_3_ concentrations to identify the threshold for a surge in the level of NM particles.

In essence, NAD blends exhibit significantly different PNSD characteristics, specifically showing a lower total PN but higher NM distribution peaks. This is attributed to the enhanced heat transfer and more complete combustion triggered by nano-Al_2_O_3_,^24^ which facilitates the oxidation of larger particles into finer particles. However, fine particles, featuring extensive specific surface areas and thus tending to facilitate the adherence of toxic substances,^58^ are often regarded as presenting a greater health risk.

**Figure 8:**
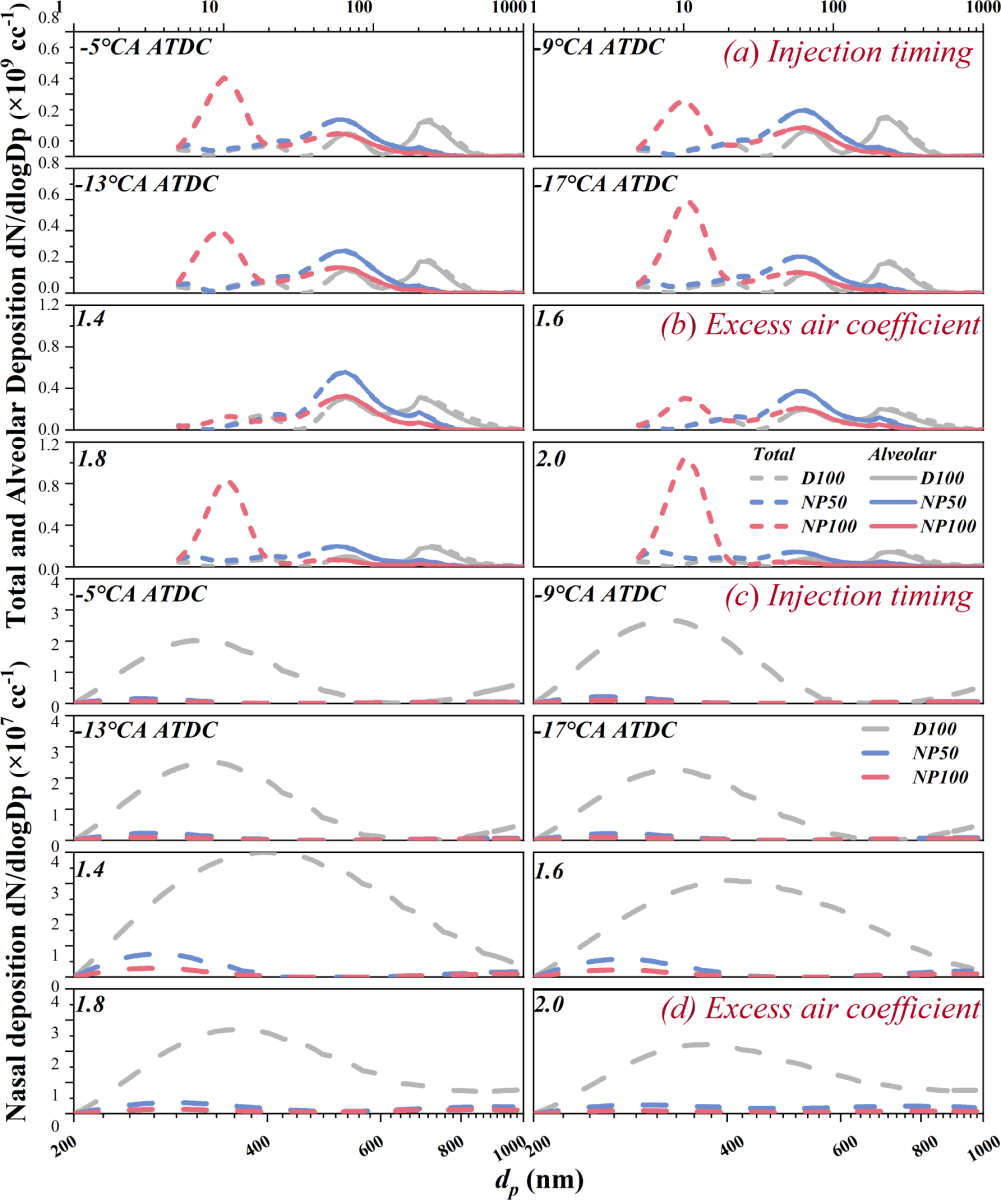
Effects of nano-Al_2_O_3_ on the total and alveolar deposition at various (a) injection timings and (b) excess air coefficients and nasal deposition at various (c) injection timings and (d) excess air coefficients.

Given this, the deposition rates of nasal breathing were employed to evaluate the effect of the changed PNSD distribution on the human body, specifically including total, alveolar, and nasal deposition. The detailed PN deposition distribution under multiple operational conditions is demonstrated in Figure 8. The PN deposition results highlight the high total PN deposition of NP100 within the range of 5–20 nm, attributed to its PN peak at 10 nm. It also indicates the high total and alveolar deposition for D100 and NAD blends within the range of 50–400 nm, with distribution peaks corresponding to their PN distributions. Additionally, the observed results spotlight the nasal deposition of D100 within the range of 200–800 nm, featuring a concentration an order of magnitude smaller due to the lower deposition rate, whereas NAD blends have virtually no nasal deposition.

Different regional depositions may induce different health effects. Typically, alveolar deposition poses a greater health threat than nasal deposition, as alveolar deposition can be absorbed by the blood, slowly cleared by the lymphatic circulation, and lead to long-term negative health effects.^59^ In this study, due to the decreasing GMD, the alveolar deposition for NAD blends is primarily within the 50–100 nm range, with NP50 having a higher peak, rather than the 100–400 nm range for D100. Therefore, there is a health risk of alveolar deposition for NP50 and a health risk of total deposition within the range of 5–20 nm for NP100, even if the nasal deposition for both had almost disappeared.

#### 3.3.3 The Diesel Engine Inherent and Emerging Trade-Off Dilemma

##### 3.3.3.1 Perennial Soot-NOx and PN-NOx Trade-Off

**Figure 9:**
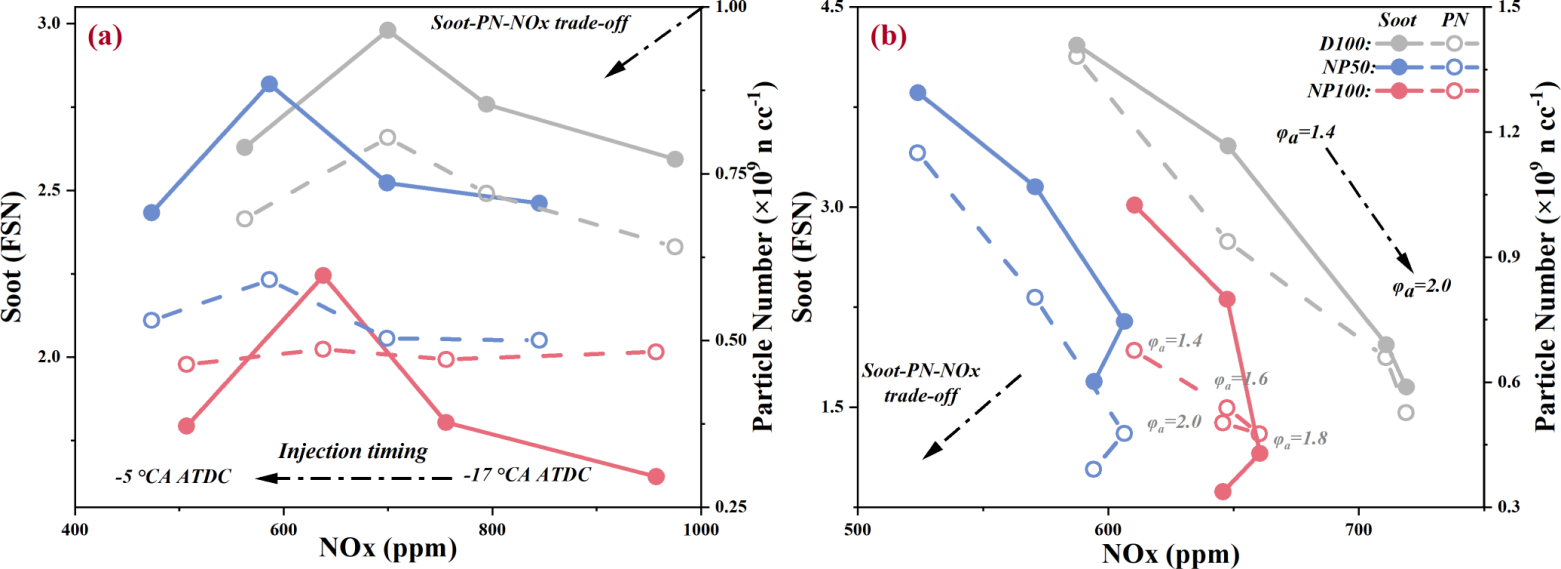
Effects of nano-Al_2_O_3_ on soot-PN-NOx trade-off at various (a) injection timings and (b) excess air coefficients.

Managing the soot-PN-NOx trade-off in DEs presents a formidable challenge due to the distinct temperature and oxygen concentration requirements for the formation of these emissions. An exploration of this trade-off, considering various IT, φ_a_, and nano-Al_2_O_3_ concentrations, is depicted in Figure 9. The study reveals that while late injection strategies curtail NOx emissions, they inadvertently elevate soot and PN emissions. Conversely, increasing φ_a_ effectively diminishes soot and PN emissions but at the expense of heightened NOx emissions. These findings underscore the potential of optimizing combustion control strategies—namely, late injection coupled with increased φ_a_ to collaboratively manage soot-PN-NOx emissions in DEs.

Nano-Al_2_O_3_ also emerges as a viable solution to the soot-PN-NOx trade-off. By facilitating heat and mass transfer, NAD blends contribute to the cooperative control of soot, PN, and NOx emissions. This synergistic effect is attributed to the nano-Al_2_O_3_’s capacity to mitigate NOx, PN, and soot formation by homogenizing the temperature distribution and the air-fuel mixture within the combustion chamber. Notably, while NAD blends demonstrate a significantly reduced total PN concentration, they also exhibit a finer GMD and an elevated concentration of nucleation mode particles, resulting in increased PN deposition in the 5–100 nm range. Consequently, the integration of nano-Al_2_O_3_ is recommended as a strategic approach to addressing the soot-PN-NOx trade-off challenge in DEs, although managing the implications of finer particle emissions remains essential.

##### 3.3.3.2 Emerging Energy Performance and Emissions Trade-Off

In the context of integrating nano-Al_2_O_3_ which has led to decreased energy performance but improved emissions, evaluating the acceptable threshold of the usage of nano-Al_2_O_3_ is crucial. With reference to Kegl and colleagues,^13^ a factor *ξ*_*A*_, which considers engine performance and emission characteristics, is employed and adjusted to assess this threshold. Specifically, the weighting factors are equally assigned to energy performance and emissions to achieve a balanced evaluation with further equal distribution among each parameter within energy performance and emissions. When *ξ*_*A*_ > 0, the incorporation of nano-Al_2_O_3_ is beneficial to the engine, whereas *ξ*_*A*_ < 0 indicates that the incorporation of nano-Al_2_O_3_ is not beneficial. The corrected criterion is defined as
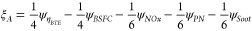
2where Ψ represents the variations of NAD blends compared to pure diesel under various engine characteristics.

As listed in Table 3, the incorporation of nano-Al_2_O_3_ is beneficial to the engine in this study because *ξ*_*A*_ remains positive under any operational conditions. This finding suggested that the substantial reduction in emissions not only counterbalances the adverse effect on energy performance but also outweighs. Additionally, under identical scenarios, NP100 consistently exhibits a higher *ξ*_*A*_ than that of NP50, owing to the dramatic reduction in emissions.

**Table 3:** Value of Acceptable Threshold *ξ*_*A*_

Injection Timing (°CA ATDC)	NP50	NP100	Excess Air Coefficient	NP50	NP100
–5	0.08	0.09	1.4	0.05	0.12
–9	0.09	0.10	1.6	0.06	0.10
–13	0.07	0.10	1.8	0.04	0.10
–17	0.06	0.09	2	0.07	0.08

## 4 Conclusions

This research evaluated the effects of nano-Al_2_O_3_ on various aspects of diesel engine performance, focusing on engine characteristics and potential health impacts under varying injection timings and excess air coefficients. The results demonstrated that nano-Al_2_O_3_/diesel (NAD) blends produced a notably increased heat release peak during the initial combustion phase, leading to a more intense pressure rise rate. This effect could be managed by leveraging late injection and lean mixing, thereby optimizing combustion dynamics. On the other hand, early injection and hypoxic scenarios were observed to extend ignition delay and combustion duration, resulting in a delayed CA50 and a greater contribution of premixed combustion to the total heat release. These negative consequences could be mitigated by the inherent properties of nano-Al_2_O_3_, such as high specific surface area and thermal conductivity, which promotes improved combustion conditions. While nano-Al_2_O_3_ slightly increased brake-specific fuel consumption and decreased brake thermal efficiency, these outcomes were primarily linked to enhanced fuel spray penetration and a subsequent decrease in the cumulative heat release rate, highlighting a trade-off between combustion efficiency and fuel economy. Importantly, nano-Al_2_O_3_ addressed the soot-PN-NOx trade-off, which is a persistent challenge in diesel engines. It achieved this by promoting uniform air-fuel mixing, thereby reducing PN and soot emissions, and by facilitating faster heat transfer, enabling reduced NOx emissions even at elevated in-cylinder temperatures. Furthermore, while the total PN concentration reduced, NAD blends exhibited an increased concentration of nucleation mode particles and a decrease in particulate geometric mean diameter. Interestingly, although NAD blends eliminated nasal deposition, there was an unignored increment of total and alveolar deposition.

In summary, despite minor compromises in energy performance due to incompatibility with existing combustion chamber dynamics, nano-Al_2_O_3_ significantly mitigated the soot-PN-NOx trade-off. This positions nano-Al_2_O_3_ as a promising additive for diesel fuels, balancing environmental considerations and energy efficiency, particularly under challenging air-fuel mixing conditions. However, future research should address the removal of nucleation mode particles with high deposition rates during in-cylinder combustion to further optimize its benefits for human health and the environment. Long-term studies on nano-Al_2_O_3_’s effects on engine components and after-treatment systems are also essential to fully assess its impact on diesel engine economics and sustainability.

## Nomenclature

Abbr.: Full name
Al_2_O_3_: aluminum oxide
AM: accumulation mode
ATDC: after the top dead center
BSFC: brake-specific fuel consumption
BTE: brake thermal efficiency
CDT: combustion duration time
DE: diesel engine
*d*_p_: particle diameter
FSN: filter smoke number
GMD: geometric mean diameter
HRR: heat release rate
IDT: injection delay time
IT: injection timing
NAD: nano-Al_2_O_3_/diesel
MCC: mixing controlled combustion
MCP: main combustion phase
NM: nucleation mode
NPs: nanoparticles
NOx: nitrogen oxides
PC: postcombustion
PCP: postcombustion phase
PRR: pressure release rate
PM: particulate matter
PN: particulate number
PPC: partially premixed combustion
PNSD: particulate number-size distribution
RCP: rapid combustion phase
RHR: ratio of heat release
φ_a_: excess air coefficient
°CA: crank angle
